# IL-23 skin and joint profiling in psoriatic arthritis: novel perspectives in understanding clinical responses to IL-23 inhibitors

**DOI:** 10.1136/annrheumdis-2020-218186

**Published:** 2020-11-26

**Authors:** Alessandra Nerviani, Marie-Astrid Boutet, Wang Sin Gina Tan, Katriona Goldmann, Nirupam Purkayastha, Tamas Ajtos Lajtos, Rebecca Hands, Myles Lewis, Stephen Kelly, Costantino Pitzalis

**Affiliations:** 1 Centre for Experimental Medicine and Rheumatology, William Harvey Research Institute, Barts and The London School of Medicine and Dentistry, Queen Mary University of London, London, UK; 2 Rheumatology Department, Mile End Hospital, Barts Health NHS Trust, London, UK

**Keywords:** arthritis, psoriatic, synovitis, biological therapy

## Abstract

**Objectives:**

To determine the relationship between synovial versus skin transcriptional/histological profiles in patients with active psoriatic arthritis (PsA) and explore mechanistic links between diseased tissue pathology and clinical outcomes.

**Methods:**

Twenty-seven active PsA patients were enrolled in an observational/open-label study and underwent biopsies of synovium and paired lesional/non-lesional skin before starting anti-tumour necrosis factor (TNF) (if biologic-naïve) or ustekinumab (if anti-TNF inadequate responders). Molecular analysis of 80-inflammation-related genes and protein levels for interleukin (IL)-23p40/IL-23p19/IL-23R were assessed by real-time-PCR and immunohistochemistry, respectively.

**Results:**

At baseline, all patients had persistent active disease as per inclusion criteria. At primary end-point (16-weeks post-treatment), skin responses favoured ustekinumab, while joint responses favoured anti-TNF therapies. Principal component analysis revealed distinct clustering of synovial tissue gene expression away from the matched skin. While *IL12B, IL23A* and *IL23R* were homogeneously expressed in lesional skin, their expression was extremely heterogeneous in paired synovial tissues. Here, IL-23 transcriptomic/protein expression was strongly linked to patients with high-grade synovitis who, however, were not distinguishable by conventional clinimetric measures.

**Conclusions:**

PsA synovial tissue shows a heterogeneous IL-23 axis profile when compared with matched skin. Synovial molecular pathology may help to identify among clinically indistinguishable patients those with a greater probability of responding to IL-23 inhibitors.

**Key messages**

**What is already known about this subject?**

- Psoriatic arthritis (PsA) is a chronic heterogeneous inflammatory condition affecting patients with psoriasis, and the interleukin (IL)-23/IL-17 axis is believed to be key in psoriasis and PsA pathogenesis.
- Several drugs targeting the IL-23/IL-17 axis have been successfully tested in the context of psoriasis and PsA but, while 50%–60% of patients achieve almost complete psoriasis clearance on treatment, the joint disease improvement is modest. To date, the mechanism for the divergent skin-joint response remains largely unexplained.

**What does this study add?**

- It provides first-time detailed evidence of the expression of the IL-23 axis in matched skin and synovial tissue from active PsA patients demonstrating distinct gene expression clustering of the synovium away from paired skin. It reveals that, while *IL23A, IL12B* and *IL23R* are expressed at a high level in lesional skin, their expression in the synovium is hugely heterogeneous.
- It demonstrates that, while patients with diverse degrees of synovial inflammation could not be distinguished clinically by conventional clinimetric measures, the IL-23 axis signature is differentially expressed within the synovial tissue and strongly linked to high-grade synovitis.

**Key messages**

**How might this impact on clinical practice or future developments?**

- This study demonstrates that psoriatic arthritis synovial tissue shows a heterogeneous interleukin 23 (IL-23) axis profile independently of its expression in paired-skin samples, thus providing a plausible mechanistic explanation for the divergent skin and joint clinical response to IL-23 inhibitors. It supports the need to test in larger appropriately designed and powered studies whether drug-target bioavailability correlates with the likelihood of response. Identifying biomarkers of joint response to therapy in patients clinically indistinguishable is going to be vital to improve disease outcomes, prevent disability and reduce healthcare and societal costs.

## Introduction

Psoriatic arthritis (PsA) is a chronic heterogeneous inflammatory condition occurring in up to 30% of patients with skin and/or nail psoriasis (PsO), which variably affects the spine, peripheral synovial joints and entheses.^1^ Although the mechanisms for such disease heterogeneity are not entirely clear, the interleukin (IL)-23/IL-17 axis is believed to be key in PsO and PsA pathogenesis.^2 3^

IL-23 is a proinflammatory cytokine composed of two subunits (p40, in common with IL-12, and p19, IL-23-specific) and mostly produced by keratinocytes, dendritic and myeloid cells. By binding its cognate receptors (IL-23R/IL-12Rβ1), it stabilises RAR-related-orphan-receptor-gamma-t (RORγt) in T-helper-17 cells, which, in turn, release their effector cytokines IL-17, IL-21 and IL-22 to initiate and amplify local autoimmune reactions and chronic inflammation.^2^

Several drugs targeting the IL-23/IL-17 axis have been successfully tested in PsO and PsA.^2^ For example, ustekinumab and secukinumab, inhibitors of IL-12/IL-23p40 and IL-17A respectively, are recommended as a second-line biological treatment for PsA patients inadequate responders to conventional-synthetic (cs) disease-modifying antirheumatic drugs (DMARDs) who had failed at least one tumour necrosis factor (TNF) inhibitor (TNFi).^4 5^ However, by blocking these pathways, while 47%–64% of patients achieve a 75%-improvement in skin disease (Psoriasis Area and Severity Index (PASI75)), success in treating joints is more modest, and a mere 20% improvement (American College of Rheumatology (ACR20)) is observed in 35%–50% of patients.^6 7^ The new IL-23p19 selective inhibitors have been shown to be more effective, and ACR20 is reached in approximately 60%.^8 9^ However, while a similar proportion of patients achieve almost complete PsO clearance (PASI90), high hurdles joint disease ACR50/ACR70 is achieved in only 33%–36% and 13%–20% of patients, respectively.^8 9^

To date, the mechanism for such divergent skin-joint response, consistent across multiple trials, remains largely unexplained. Boutet *et al* 2 and Belasco *et al* 10 have postulated that different target expression levels in skin and joints contribute to the diverse clinical response. For example, Belasco *et al* reported that gene expression patterns in skin and synovium are distinct, showing a stronger IL-17 signature in skin than in synovium, and more equivalent TNF signal across both tissues.^10^ Here, we present new evidence exploring the expression of the IL-12/IL-23 axis in psoriatic skin versus matched synovial tissue at both molecular and protein level.

## Methods

Full methods are included in online supplemental material. Briefly, 27 patients fulfilling the Classification Criteria for Psoriatic Arthritis (CASPAR)^11^ with active peripheral joint disease despite csDMARDs and either biologic-naïve/ failing TNFi were recruited in this observational/open-label study (REC15/LO/0584). Patients underwent a baseline ultrasound (US)-guided synovial biopsy^12^ and lesional/non-lesional skin punch-biopsies, and were then treated with TNFi/ustekinumab as per local guidelines. The chosen primary endpoint was 16 weeks. Gene expression was analysed by real-time PCR (Fluidigm). Paraffin-embedded skin/synovium samples were stained with H&E. Immune cells/IL-23-axis were quantified by immunohistochemistry. Synovial tissue were categorised in ‘low-grade’(score 0–1) or ‘high-grade’(score 2–7) synovitis^13^ and in pathotypes (lympho-myeloid/diffuse-myeloid/pauci-immune).^14^

10.1136/annrheumdis-2020-218186.supp1 Supplementary data

## Results

### Patients’ characteristics and treatment response

**Figure 1:**
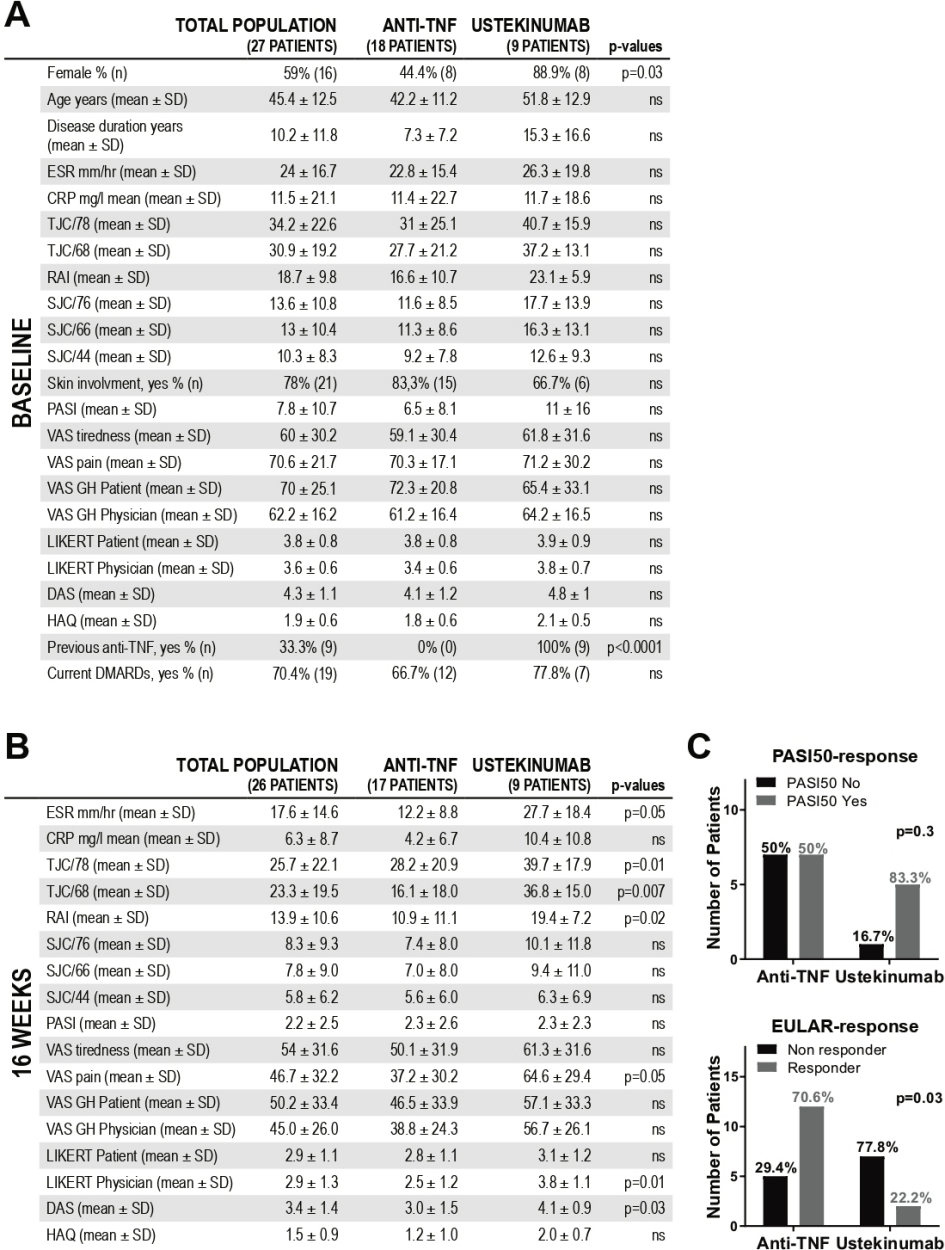
Baseline and 16 weeks characteristics of the patients included in the psoriatic arthritis pathobiology and its relationship with clinical disease activity (PsABRE) study. (A) Baseline features of the whole cohort (n=27) and comparison of variables between patients receiving anti-TNF (n=18) or ustekinumab (n=9). (B) Patients’ characteristics at the chosen primary endpoint, that is, 16-weeks post-treatment (n=26, one patient lost to follow-up) and comparison between TNFi- (n=17) and ustekinumab-treated patients (n=9). (A, B) P values calculated using Mann-Whitney U test or Fisher’s exact test as required (TNFi-arm vs ustekinumab-arm). (C) Skin (PASI50) and joints (EULAR(DAS) good/moderate vs none) response at 16 weeks. P values calculated using Fisher’s exact test. CRP, C reactive protein; DAS, Disease Activity Score; DMARDs, disease-modifying antirheumatic drugs; ESR, erythrocyte sedimentation rate; N, number; NS, non-significant; PASI, Psoriasis Area and Severity Index; RAI, Ritchie Articular Index; SJC, swollen joints count; TJC, tender joints count; TNF, tumour necrosis factor; VAS, Visual Analogue Scale (0–100).

Baseline and 16 weeks demographic and clinical features are summarised in figure 1. The overall male to female ratio was ~1:1 (59% female), the average age was 45.4±12.5 and disease duration >10 years. Seventy-eight per cent of patients had concomitant skin involvement, with a mean PASI of 7.8. As per inclusion criteria, all patients had active joint disease (68-tender joints count 30.9±19.2, 66-swollen joints count 13±10.4, Disease Activity Score (DAS) 4.3±1.1) despite treatment with csDMARDs±anti TNF. Following the baseline biopsy, patients were treated with anti-TNF (n=18) if they were biologic-naïve or ustekinumab (n=9) if they had not responded to at least one TNFi. The higher number of females in the ustekinumab-arm (8/9) reflects the gender differences in TNFi-treatment outcomes observed in registries^15^ (figure 1A). At 16 weeks, ESR, tender-joint scores, Ritchie Articular Index (RAI), Visual Analogue Scale (VAS)-pain, Likert-physician-assessment and DAS were significantly higher in the ustekinumab-treated group; PASI-scores improved from baseline in both groups (−4.7±7.5 in TNFi treated vs −8.9±14.3 in ustekinumab treated) and were comparable between the two treatment arms (2.3±2.6 in TNFi treated vs 2.3±2.3 in ustekinumab treated) (figure 1B). However, while significantly more patients in the anti-TNF group achieved EULAR(DAS)-response compared with ustekinumab-treated patients (70.6% vs 22.2%), there was a trend in favour of ustekinumab in terms of skin responses (figure 1C). Besides, as joint response to ustekinumab can be delayed up to 24–28 weeks, clinical responses were also assessed at 24 weeks. As shown in online supplemental figure S1, ustekinumab-treated patients maintained significantly higher tender joint scores, RAI, VAS-pain, Likert physician assessment and DAS; 50% and 68.8% of patients in the ustekinumab and TNFi arms achieved EULAR(DAS) response, respectively. Individual patient joint/skin responses are summarised in online supplemental table S1.

### Gene expression profiles in paired skin and synovium reveal tissue-specific signatures and divergent expression patterns

**Figure 2:**
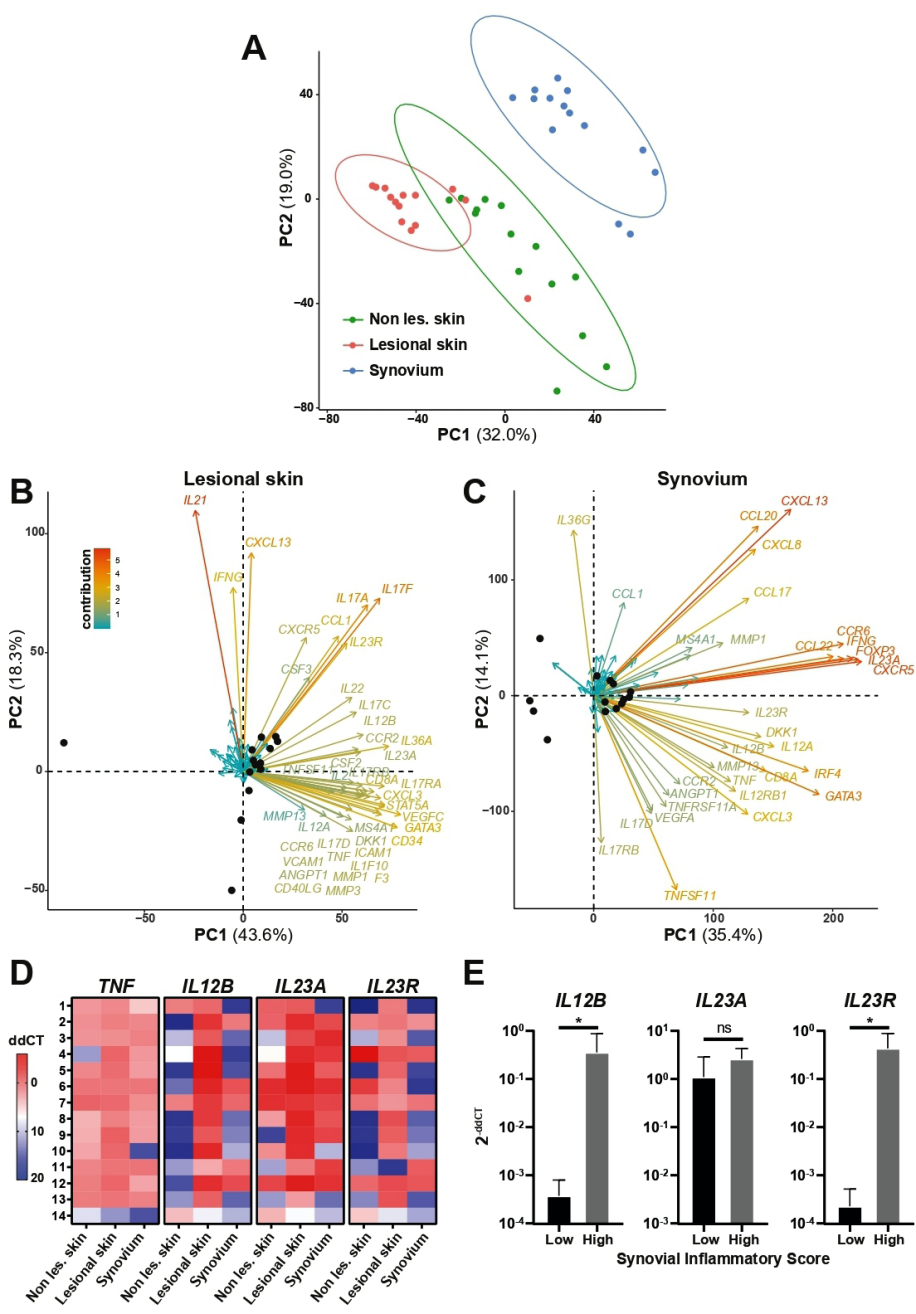
Gene expression analysis in matched skin and synovium from PsA patients. (A) Principal component (PC) analysis (PCA) performed on the expression data of a set of 80 selected genes in 14 matched non-lesional (non les.) and lesional skin and synovium. The first two eigenvalues were plotted with data ellipses for each tissue type using a CI of 0.95. The PCA clearly separates synovium (blue dots) from non-lesional skin (non les., green dots) and lesional skin (red dots). (B, C) Biplots showing individuals repartition in PC1 and 2 (black dots) and loading plots assessing the contribution of each of the 80 genes analysed in the PC, displayed for the lesional skin (B) and synovial tissues (C). Genes names are indicated if their contribution to the PC variance is >1. The PCA and biplots were created using function prcomp from the stats package within R statistics (version 3.5.3) and factoextra R package^20^ (D) Heatmap representing *TNF*, *IL12B* (IL-23p40 protein), *IL23A* (IL-23p19 protein) and *IL23R* expression in 14 matched non-lesional (non les.) and lesional skin and synovium samples. dd-threshold cycles (ddCTs) are shown in colorimetric scale (low expression in blue, high expression in red). Lines 1–11 represent anti-TNF-treated patients, lines 12–14 ustekinumab-treated patients. E, *IL12B*, *IL23A* and *IL23R* gene expression in synovial biopsies classified as ‘low’ (0–1) and ‘high’ (2–7) synovial inflammatory score (Krenn’s score). P values were calculated using Mann-Whitney U test, *P<0.05, mean and SD are shown. IL-23, interleukin 23; TNF, tumour necrosis factor.

Gene expression analysis was performed on 14 matched synovial tissue, lesional and adjacent non-lesional skin. As shown in figure 2A, principal component analysis (PCA), built on the expression of 80 inflammation-related genes (online supplemental table S2), showed that the synovium clusters away from the skin, with a partial overlapping of lesional and non-lesional skin. To further investigate the gene variance contributing to the diversity of expression within each anatomic site (skin/synovium), related PCA plots were covisualised with loading plots (biplots) (figure 2B and C). *IL17A/F*, *IL23R* and *IL21* were the major contributors of PC1/2 variation in lesional skin. In synovium, genes related to ectopic lymphoid structure (ELS) formation (*CXCL13*, *CXCR5*) and the IL-23 axis (*IL23A*, *IL12B, IL23R*) together strongly contributed to the PC variation. For instance, *CXCR5* and *IL23A* robustly aligned with PC1 in accounting for 35.4% of the variance within the synovium data set and *CXCL13* strongly and equally contributed to PC1 and PC2 variation. We next assessed the relative gene expression of the drug-targets of TNF- and IL-23/IL-12-inhibitors, that is, *TNF*, *IL23A* (encoding IL-23p19), *IL12B* (encoding IL-23p40) and *IL23R* (figure 2D). *TNF* was generally homogeneously expressed in both skin and synovial tissue. Conversely, *IL23A*, *IL12B* and *IL23R* showed higher expression in lesional skin compared with both non-lesional skin and synovium. Interestingly, we observed that while some patients did express IL-23 cytokines/receptor in both skin and joint, others had discordant expression, that is, active IL-23 pathway in the lesional skin but not in the synovium. To investigate potential mechanisms for the diverse expression of the IL-23-axis within the synovium, we stratified patients based on the degree of synovial inflammation.^13^ Both *IL12B* and *IL23R* genes, but not *IL23A*, were significantly more expressed in patients with higher synovitis scores (figure 2E). Notably, despite the major variance in the degree of synovial inflammation and histological pathotypes, there were no significant clinical differences in the two patient groups (online supplemental table S3, S4).

### Synovial IL-23p40/p19 and IL-23R protein expression correlates with the histological inflammatory status

**Figure 3:**
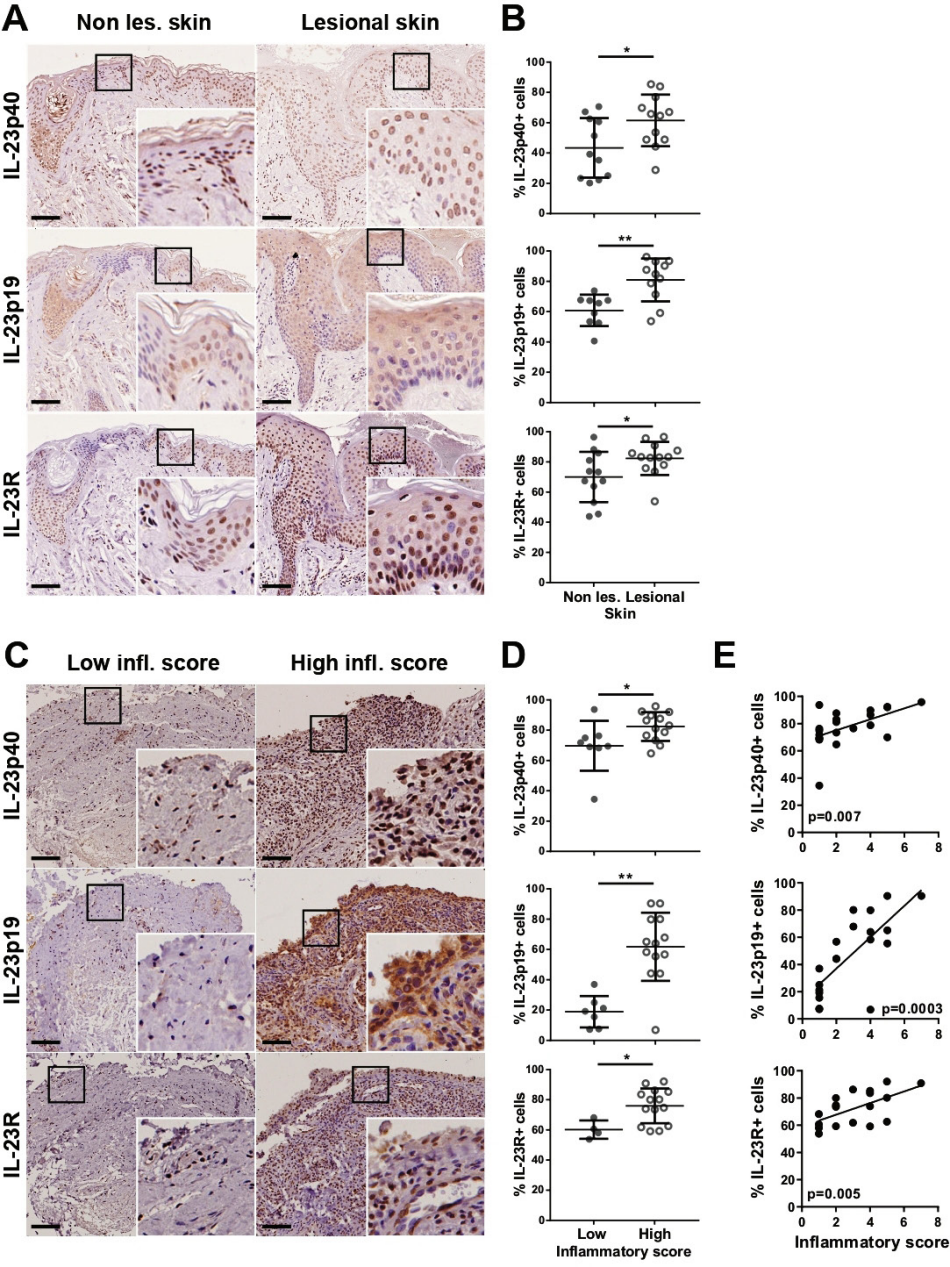
Expression of IL-23p40, IL-23p19 and IL-23R in skin and synovium from PsA patients. (A, C) representative images of sections of PSA non-lesional (non les.) and lesional skin (A) and synovial tissue of different degree of inflammatory scores (C) immunostained for IL-23p40, IL-23p19 and IL-23R. Scale bar=200 µm. Enlarged images correspond to the respective boxed areas. (B, D) Digital image analysis was performed on non-lesional and lesional skin (B) (n=11–12) and synovium (D) (low inflammatory score, n=4–8; high inflammatory score, n=13–14) sections. IL-23p40, IL23p19 and IL23R positive cells were determined using QuPath software^21^ and are presented as % of the total number of cells. Results are shown as mean±SD. *P<0.05, **P<0.01 as assessed by Mann-Whitney U test. (E) Correlations between inflammatory scores and IL-23p40, IL-23p19 or IL-23R percentages of positive cells within the synovial tissue. P values, calculated by Spearman’s bivariate correlation analysis, are indicated on each graph. IL-23, interleukin 23; PsA, psoriatic arthritis.

To confirm the molecular findings, we next evaluated protein expression levels of IL-23p40, IL-23p19 and IL-23R in skin and synovium by immunohistochemistry. As expected, the percentage of IL-23p40-, IL-23p19- and IL-23R-positive cells was significantly higher in lesional skin compared with paired non-lesional skin (figure 3A, B); within the synovium, it was greater in patients with higher degree of inflammation (figure 3C, D) and in lympho-myeloid and diffuse-myeloid pathotypes (online supplemental figure S2). This result was in line with the positive correlation observed between the synovial inflammatory score and the proportion of IL-23p40/IL-23p19/IL-23R-positive cells (figure 3E), as well as their correlation with each other’s (data not shown). Of note, the percentage of IL-23p40/IL-23p19/IL-23R-positive cells at baseline was, on average, comparable between the treatment groups despite different drug exposure (online supplemental table S5). Except for the LIKERT patient score, we did not detect other significant correlations between IL-23-axis expression and clinical parameters at baseline, suggesting that patients with comparable disease severity may have, in fact, heterogeneous histopathological features and expression of drug targets within the diseased synovium (online supplemental table S6).

To further assess whether the IL-23-axis heterogeneity tracks across different stages of the disease, we analysed IL-23 expression pattern in the synovium of 21 treatment-naïve PsA patients with <12 months symptoms. As shown in online supplemental figure S3, overall, there was a positive correlation between IL-23p40/IL-23p19/IL-23R-positive cells and synovitis scores, and lower IL-23 cytokines/receptor tissue-availability in the pauci-immune compared with macrophage-rich pathotypes. Similarly to established PsA, we did not find significant correlations between clinical parameters and IL-23 axis expression. Finally, to investigate whether the differential IL-23-expression observed in PsA synovium was disease-specific or related to synovial histopathology, we quantified IL-23p40/IL-23p19/IL-23R in a cohort of 17 treatment-naïve rheumatoid arthritis (RA) patients spanning diverse degrees of synovial inflammation and histopathotypes, and confirmed that, at least in the early phases of RA, IL-23 expression pattern is pathology related and significantly associates with the presence of ELS (online supplemental figure S4).

## Discussion

To our knowledge, this study provides first-time detailed evidence of the expression of the IL-23 axis (IL-23p40/IL-23p40p19/IL-23R), both at transcript and protein level, in matched skin-synovium obtained from clinically active PsA patients before undergoing anti-TNF or ustekinumab.

Using a PCR-Fluidigm-assay of 80 inflammation-related genes, first, we demonstrated distinct synovial gene expression clustering away from paired skin but a partial overlapping between lesional and non-lesional skin profiles. We also showed that IL-17 and IL-23 cytokines together with CXCL13/CXCR5, key chemokines involved in ELS formation, significantly contribute to the gene expression variance within skin and joint sites, respectively. These results are in line with those reported by Belasco *et al* 10 demonstrating that IL-17 is a major contributor of the gene expression variability within the lesional skin, and Celis *et al* 16 who showed that in synovial biopsies (unmatched for skin samples) the expression of IL-23 correlates with ELS-positive samples.

The analysis of the expression profiles of biological DMARDs targets demonstrated that *TNF* was more homogeneously expressed in skin and synovial tissue, while *IL23A*/*IL12B*/*IL23R* were generally higher-expressed in lesional skin compared with both non-lesional skin and synovium. The synovial expression of *IL23A/IL12B/IL23R* was, in fact, greatly heterogeneous and could be either similar to or much lower than the paired lesional skin. Notably, *IL12B* and *IL23R* transcripts levels were dependent on the degree of tissue inflammation, being more expressed in the presence of higher synovitis scores. Similarly, we confirmed a preferential expression of IL-23p40/IL-23p19/IL-23R proteins in patients with high-grade synovitis and immune-cells-rich histopathotypes. Importantly, patients with variable degrees of synovial inflammation and diverse pathotypes, as well as different levels of IL-23-cytokines/receptor could not be phenotypically distinguished by conventional clinical scores. Furthermore, despite variable drug exposure, the pathology of the IL-23 axis in active patients was comparable at baseline. We confirmed that IL-23-axis expression relates to the synovial histopathology not only in PsA at different stages of the disease, including early treatment-naïve patients, but also in the early phase of RA, investigated as disease control. Therefore, the pattern of expression of the IL-23 axis does not seem to be disease-specific but rather dependent on the inflammatory status and histological features of the synovial tissue in both PsA and RA.

While it is generally accepted that patients with high disease activity respond better to biologics, clinimetric measures cannot determine the grade of histological synovitis or drug-target expression levels. Tissue bioavailability of the ‘target’, of course, does not guarantee clinical response; however, there is evidence to suggest that, for example, TNF levels in RA synovium are associated with better response to TNFi,^17^ and other specific synovial tissue signatures are linked with different outcomes to anti-TNF^18^ and anti-IL-6R therapy.^19^ The results reported here support the concept that heterogeneous drug target bioavailability in the diseased tissue might also apply to the IL-23 axis. This prompted the hypothesis that different joint response rates in PsA, often divergent from the skin-response, might be explained, at least partially, by the preferential expression of the IL-23-axis by subsets of patients with higher histological synovitis but not necessarily higher disease activity.

PsABRE was an exploratory study, not designed to assess efficacy; thus, the relatively small sample size in each treatment-arm did not allow to test the above hypothesis. Moreover, no direct comparisons could be carried out between the anti-TNF- and the ustekinumab-treated cohorts: both populations failed to respond to csDMARDs, but while the former was biologic-naïve, the latter had inadequately responded to at least one TNFi representing, therefore, a more difficult-to-treat group. The trial took place in a real-life setting with no external or industry support; hence, the recruitment and treatment allocation had to follow the UK National Institute for health and Care Excellence prescription guidelines with consequent different drug exposure in the two groups. Despite these limitations, the main value of the study resides in its molecular pathology characterisation of paired skin and US-guided synovial biopsies of the most inflamed joint, including small joints, that demonstrates a divergent profile between the two diseased tissues and, generally, a lower level of expression of the IL-23 axis in the synovial tissue particularly in patients with low-grade synovitis.

The heterogeneous synovial expression of the IL-23-axis provides a plausible mechanistic explanation for the divergent outcomes consistently observed in clinical trials whereby IL-23i have better results in PsA skin than in joints. This hypothesis needs to be tested in larger, appropriately designed and powered studies. Identifying biomarkers of joint-response to therapy in patients clinically indistinguishable is going to be vital to refine PsA clinical classification and enrich for treatment response while reducing unnecessary exposure to costly and potentially toxic medications.

## Data Availability

Data are available on reasonable request. All data relevant to the study are included in the article or uploaded as online supplemental information.
