# Supplementary material for: IL-23 skin and joint profiling in psoriatic arthritis: novel perspectives in understanding clinical responses to IL-23 inhibitors
Source: Ann Rheum Dis. 2020 Nov 26;80(5):591–7. doi: 10.1136/annrheumdis-2020-218186 (PMC8053336; doi:10.1136/annrheumdis-2020-218186)
Supplement: Supplementary data [file annrheumdis-2020-218186supp001.pdf]

SUPPLEMENTARY METHODS

Patients

Twenty-seven (27) patients fulfilling the ClASsification for Psoriatic ARthritis (CASPAR) criteria [11] with active peripheral joint disease (≥3 tender and ≥3 swollen joints) despite an adequate trial of at least two csDMARDs and either biologic-naïve or failing one or more TNFα-inhibitor (TNFi) were recruited to this observational/open-label real-life study **Ps**oriatic **A**rthritis Patho**B**iology and its **R**elationship with Clinical Diseas**E** Activity (PsABRE) at Bart's Health NHS Trust. All patients underwent a baseline ultrasound (US)-guided needle synovial biopsy of the most inflamed peripheral joint, including small joints, as previously described.[12] Lesional and adjacent non-lesional skin punch biopsies were collected from patients with active skin disease at baseline. According to the National Institute for Health and Care Excellence (NICE UK) prescribing guidelines, patients biologic-naïve were treated with TNFi while patients who had already failed one or more anti-TNF agents received ustekinumab. The chosen primary endpoint was 16-weeks. Clinical and response data were also collected at 24-weeks in view of the delayed response to ustekinumab. Summary of treatments and associated EULAR/PASI50 responses at 16 and 24-weeks are presented in **Supplementary Table.S1**. Synovial/skin biopsy samples were partly stored in RNA-later and partly fixed in formalin. All patients gave written informed consent prior to recruitment. The study was approved by the local ethics committee (REC 15/LO/0584).

To validate the relationship between the IL-23-axis expression and the synovial histopathology, 21 psoriatic arthritis and 17 rheumatoid arthritis patients part of the Pathobiology of Early Arthritis Cohort (05/Q070/198) were also analysed. All patients had <12 months duration of symptoms and were treatment-naïve when underwent US-guided synovial biopsy.

Patient and Public Involvement statement

No funding or time were specifically allocated to patient and public involvement (PPI) in the original application. However, a patients' survey was conducted at the time of the trial to ensure patients were satisfied with the care received as part of the research study and to receive feedback to improve the delivery of the study.

Gene expression analysis

Total RNA was extracted from synovium and skin using a Trizol/Chloroform method as previously described.[14] The relative expression of 80 genes relevant to inflammatory pathways, including genes encoding IL-23-cytokines/receptor, was quantified by real-time PCR using Fluidigm technology (Fluidigm corporation, San Francisco, CA-USA) (**Supplementary Table.S2**). Data were analysed with a

Nerviani A, *et al. Ann Rheum Dis* 2021; 80:591–597. doi: 10.1136/annrheumdis-2020-218186

ΔΔCt method using beta-glucuronidase (GUSB) as the housekeeping gene and a mix of cDNA from 3 synovial-tissue samples as reference.

Histology, immunohistochemistry and image analysis

Skin specimens (lesional/non-lesional) and a minimum of 6 synovial-tissue fragments were paraffin-embedded and sectioned at 3µm. Synovial samples were stained with Haematoxylin and Eosin (H&E), and the presence/degree of synovitis was quantified based on parameters previously published [13] by two independent observers; samples were defined as "low-grade synovitis" if score 0-1 or "high-grade synovitis" if score 2-7. Moreover, as previously described in RA synovial tissue [14], synovial tissue sections were immuno-stained for CD3, CD20, CD68 and CD138 in order to quantify the immune infiltrate of T-cells, B-cells, macrophages and plasma cells, respectively. Based on a combination of semi-quantitative score [14], patients were categorised in histological pathotypes (lympho-myeloid, diffuse-myeloid and fibroid/pauci-immune) as previously described [14]. To assess the presence and distribution of IL-23-related cytokines and receptors-(R), both synovial and skin specimens were immuno-stained for IL-23p40 (Novus Biologicals, Centennial, CO-USA), IL-23p19 (BioLegend, San Diego, CA-USA) and IL-23R (Novus Biologicals). Matching isotype antibodies were used as controls. IL-23R cell specificity has also been tested by double immunofluorescence staining with CD3, CD68 and CD20 (data not shown). Slides were counterstained with Haematoxylin and mounted with DPX mounting medium (Sigma-Aldrich, Saint-Louis, MO-USA). All sections were scanned using the digital slide scanner Nanozoomer S210 (Hamamatsu Photonics, Japan). The percentage of positive cells was determined by quantitative digital image analyses using QuPath software.[20]

Statistical analysis

Differences in continuous variables were analysed by Mann–Whitney U-test (two groups) or Kruskal–Wallis with Dunn's post-test (multiple groups). Fisher's exact test was used to evaluate significant associations between categorical variables. Correlations were analysed by Spearman's correlation test. Statistical analyses were performed using GraphPad-Prism-v8-software, p-values<0.05 were considered significant. The PCA and biplots were created using function prcomp from the stats package within R statistics (version 3.5.3) and factoextra R package.[21] The first two eigenvalues were plotted with data ellipses for each tissue type using a confidence interval of 0.95.

Supplementary Figure S1

A

24 WEEKS	TOTAL POPULATION (n=24)	ANTI-TNF (n=16)	USTEKINUMAB (n=8)	p-values
ESR mm/hr (mean ± SD)	14.8 ± 13.0	11.6 ± 10.0	21.3 ± 16.4	ns
CRP mg/l mean (mean ± SD)	4.8 ± 7.7	3.8 ± 6.3	7.0 ± 10.1	ns
TJC/78 (mean ± SD)	23.2 ± 22.6	17.6 ± 23.0	34.5 ± 18.1	p=0.03
TJC/68 (mean ± SD)	21.0 ± 19.6	16.1 ± 20.0	30.9 ± 15.3	p=0.03
RAI (mean ± SD)	11.8 ± 10.1	8.4 ± 8.9	18.6 ± 9.4	p=0.01
SJC/76 (mean ± SD)	6.2 ± 8.4	4.5 ± 7.4	9.5 ± 9.9	ns
SJC/66 (mean ± SD)	5.5 ± 7.7	4.1 ± 7.2	8.5 ± 8.2	ns
SJC/44 (mean ± SD)	4.1 ± 5.4	3.3 ± 5.6	5.9 ± 4.9	ns
PASI (mean ± SD)	2.0 ± 2.6	2.2 ± 2.7	1.6 ± 2.8	ns
VAS tiredness (mean ± SD)	52.4 ± 33.5	45.5 ± 31.2	66.3 ± 35.5	ns
VAS pain (mean ± SD)	49.1 ± 33.9	38.1 ± 31.6	71.0 ± 28.5	p=0.03
VAS GH Patient (mean ± SD)	46.8 ± 32.6	39.6 ± 29.9	61.3 ± 35.1	ns
VAS GH Physician (mean ± SD)	39.4 ± 23.8	31.8 ± 21.7	54.8 ± 21.2	ns
LIKERT Patient (mean ± SD)	3.2 ± 1.1	2.8 ± 1.0	3.9 ± 1.0	p=0.03
LIKERT Physician (mean ± SD)	2.7 ± 1.0	2.4 ± 1.1	3.3 ± 0.7	ns
DAS (mean ± SD)	2.9 ± 1.5	2.5 ± 1.5	3.9 ± 1.0	p=0.03

B

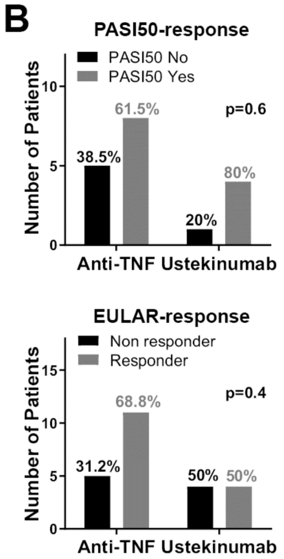

Figure S1 24-weeks characteristics of the patients included in the Psoriatic Arthritis Pathobiology and its Relationship with Clinical Disease Activity (PsABRE) study. A, Patients' characteristics at 24-weeks post-treatment (n=24, three patients lost to follow-up) and comparison between TNFi- (n=16) and ustekinumab-treated patients (n=8). p-values calculated using Mann–Whitney U-test or Fisher's exact test as required (TNFi-arm vs ustekinumab-arm). B, Skin (PASI50) and joints [EULAR(DAS) good/moderate vs none] response at 24-weeks. p-values calculated using Fisher's exact test.

SD, Standard Deviation; n, number; ESR, erythrocyte sedimentation rate; CRP, C-Reactive Protein; TJC, Tender Joints Count; SJC, Swollen Joints Count; RAI, Ritchie Articular Index; PASI, Psoriasis Area and Severity Index; VAS, Visual Analogue Scale (0-100); GH, Global Health; DAS, Disease Activity Score; TNF, Tumour Necrosis Factor; DMARDs, Disease Modifying Anti-Rheumatic Drugs; ns, non-significant; EULAR, European League Against Rheumatism.

Nerviani A, *et al. Ann Rheum Dis* 2021; 80:591–597. doi: 10.1136/annrheumdis-2020-218186

Supplementary Table S1

		EULAR-RESPONSE		PASI50-RESPONSE	
PATIENT ID	TREATMENT	16 WEEKS	24 WEEKS	16 WEEKS	24 WEEKS
PsABRE-01	Anti-TNF	1	1	0	0
PsABRE-02	Anti-TNF	0	0	N/A	NA
PsABRE-03	Anti-TNF	0	LtFU	1	LtFU
PsABRE-04	Anti-TNF	1	1	0	0
PsABRE-05	Anti-TNF	1	1	N/A	NA
PsABRE-06	Anti-TNF	1	1	1	1
PsABRE-07	Anti-TNF	1	1	1	1
PsABRE-08	Anti-TNF	LtFU	LtFU	N/A	LtFU
PsABRE-09	Anti-TNF	1	1	0	1
PsABRE-10	Anti-TNF	0	0	1	1
PsABRE-11	Anti-TNF	1	1	0	0
PsABRE-12	Anti-TNF	1	1	1	1
PsABRE-13	Anti-TNF	0	0	1	1
PsABRE-14	Anti-TNF	1	1	0	0
PsABRE-15	Anti-TNF	1	1	1	1
PsABRE-16	Anti-TNF	1	1	0	1
PsABRE-17	Anti-TNF	1	0	0	0
PsABRE-18	Anti-TNF	0	0	N/A	NA
PsABRE-19	Ustekinumab	1	LtFU	0	LtFU
PsABRE-20	Ustekinumab	0	0	N/A	NA
PsABRE-21	Ustekinumab	0	1	1	1
PsABRE-22	Ustekinumab	0	1	1	1
PsABRE-23	Ustekinumab	1	1	1	1
PsABRE-24	Ustekinumab	0	1	N/A	NA
PsABRE-25	Ustekinumab	0	0	1	0
PsABRE-26	Ustekinumab	0	0	1	1
PsABRE-27	Ustekinumab	0	0	N/A	NA

Table S1 Summary of patients’ treatment distribution (anti-TNF or ustekinumab), and individual joint [EULAR(DAS) good/moderate *versus* none] and skin (PASI50) responses.

0*, non-responder;* 1*, responder;* LtFU*, Lost to Follow-Up;* N/A*, Not Applicable;* TNF*, Tumour Necrosis Factor;* EULAR*, European League Against Rheumatism;* PASI*, Psoriasis Area and Severity Index.*

Supplementary Table S2

GENE NAME	TAQMAN PROBE	GENE NAME	TAQMAN PROBE
ANGPT1	Hs00919202_m1	*IL17D*	Hs00972161_m1
ANGPT2	Hs00169867_m1	*IL17F*	Hs01028648_m1
CCL1	Hs00234140_m1	*IL17RA*	Hs01056316_m1
CCL17	Hs00171074_m1	*IL17RB*	Hs00218889_m1
CCL19	Hs00171149_m1	*IL18*	Hs01038788_m1
CCL2	Hs00234140_m1	*IL1B*	Hs01555410_m1
CCL20	Hs00355476_m1	*IL1F10*	Hs00544661_m1
CCL21	Hs00171076_m1	*IL1R1*	Hs00991010_m1
CCL22	Hs01574247_m1	*IL2*	Hs00174114_m1
CCR2	Hs01560352_m1	*IL21*	Hs00222327_m1
CCR6	Hs00171121_m1	*IL22*	Hs01574154_m1
CD163	Hs00174705_m1	*IL23A*	Hs00372324_m1
CD28	Hs01007422_m1	*IL23R*	Hs00332759_m1
CD34	Hs02576480_m1	*IL36A*	Hs00205367_m1
CD40LG	Hs00163934_m1	*IL36B*	Hs00758166_m1
CD68	Hs00154355_m1	*IL36G*	Hs00219742_m1
CD8A	Hs00233520_m1	*IL36RN*	Hs00202179_m1
CSF1	Hs00174164_m1	*IL6*	Hs00174131_m1
CSF2	Hs99999044_m1	*IRF4*	Hs00180031_m1
CSF3	Hs99999083_m1	*MMP1*	Hs00899658_m1
CXCL12	Hs02829207_m1	*MMP13*	Hs00942584_m1
CXCL13	Hs00757930_m1	*MMP3*	Hs00968305_m1
CXCL2	Hs00601975_m1	*MMP9*	Hs00957562_m1
CXCL3	Hs00171061_m1	*MS4A1*	Hs00544819_m1
CXCL8	Hs00174103_m1	*PDPN*	Hs00366766_m1
CXCR4	Hs00976734_m1	*SOCS1*	Hs00705164_s1
CXCR5	Hs00173527_m1	*SOCS3*	Hs02330328_s1
DKK1	Hs00183740_m1	*STAT3*	Hs00374280_m1
F3	Hs01076029_m1	*STAT5A*	Hs00234181_m1
FOXP3	Hs01085834_m1	*STAT6*	Hs00598625_m1
GATA3	Hs00231122_m1	*TGFB1*	Hs00998133_m1
ICAM1	Hs00164932_m1	*TIMP1*	Hs01092511_m1
IFNG	Hs00989291_m1	*TIMP2*	Hs00234278_m1
IL10	Hs00961622_m1	*TLR4*	Hs00152939_m1
IL12A	Hs01073447_m1	*TNF*	Hs00174128_m1
IL12B	Hs01011518_m1	*TNFRSF11A*	Hs00921372_m1
IL12RB1	Hs01106578_m1	*TNFSF11*	Hs00243522_m1
IL15	Hs01003716_m1	*VCAM1*	Hs01003372_m1
IL17A	Hs00174383_m1	*VEGFA*	Hs00900055_m1
IL17C	Hs00171163_m1	*VEGFC*	Hs01099203_m1

Table S2 List of TaqMan probes used for gene expression analysis by real-time polymerase chain reaction (PCR) with Fluidigm technology (Fluidigm corporation, San Francisco, CA-USA).

Nerviani A, *et al. Ann Rheum Dis* 2021; 80:591–597. doi: 10.1136/annrheumdis-2020-218186

Supplementary Table S3

		LOW INFLAMMATORY SCORE 0-1 (9 PATIENTS)	HIGH SYNOVIAL INFLAMMATORY SCORE 2-7 (15 PATIENTS)	p-values
BASELINE	Female % (n)	33.3% (3)	73.3% (11)	ns
	Age years (mean ± SD)	42.7 ± 12.9	48.1 ± 13.0	ns
	Disease duration years (mean ± SD)	13.3 ± 12.9	8.4 ± 12.0	ns
	ESR mm/hr (mean ± SD)	19.9 ± 17.3	27.5 ± 17.2	ns
	CRP mg/l mean (mean ± SD)	10.3 ± 18.8	14.0 ± 24.4	ns
	TJC/78 (mean ± SD)	34.8 ± 19.5	32.9 ± 23.6	ns
	TJC/68 (mean ± SD)	31.2 ± 16.3	29.9 ± 20.1	ns
	RAI (mean ± SD)	18.8 ± 10.4	18.3 ± 8.8	ns
	SJC/76 (mean ± SD)	10.8 ± 7.3	14.2 ± 11.1	ns
	SJC/66 (mean ± SD)	10.6 ± 7.4	13.7 ± 11.5	ns
	SJC/44 (mean ± SD)	9.1 ± 6.4	11.1 ± 9.8	ns
	Skin involvment, yes % (n)	77.7% (7)	73.3% (11)	ns
	PASI (mean ± SD)	5.7 ± 6.2	4.0 ± 3.7	ns
	VAS tiredness (mean ± SD)	58.1 ± 29.0	62.4 ± 29.8	ns
	VAS pain (mean ± SD)	63.0 ± 17.7	73.2 ± 23.7	ns
	VAS GH Patient (mean ± SD)	66.3 ± 18.9	71.7 ± 27.1	ns
	VAS GH Physician (mean ± SD)	58.2 ± 14.4	65.5 ± 12.1	ns
	LIKERT Patient (mean ± SD)	3.8 ± 0.7	3.8 ± 0.8	ns
	LIKERT Physician (mean ± SD)	3.3 ± 0.5	3.7 ± 0.6	ns
	DAS (mean ± SD)	4.1 ± 1.1	4.5 ± 1.2	ns
	HAQ (mean ± SD)	1.8 ± 0.6	2.0 ± 0.6	ns
	Previous anti-TNF, yes % (n)	33.3% (3)	33.3% (5)	ns
	Current DMARDs, yes % (n)	55.6% (5)	73.3% (11)	ns
	Assigned to Anti-TNF, % (n)	66.7% (6)	66.7% (10)	ns
	Assigned to Ustekinumab,% (n)	33.3% (3)	33.3% (5)	ns

Table S3 Baseline clinical variables comparison between patients characterized by a lower (n=9) or higher (n=15) synovial inflammatory score [13]. 24/27 baseline synovial tissues were included in the analysis; 3 samples were classified as “ungraded” (absence of lining layer and/or necrotic tissue and/or absent of macrophages in the sublining). p-values were calculated using Mann–Whitney U-test or Fisher's exact test as appropriate.

SD*, Standard Deviation;* n*, number*; ESR*, erythrocyte sedimentation rate;* CRP*, C-Reactive Protein;* TJC*, Tender Joints Count;* SJC*, Swollen Joints Count;* RAI*, Ritchie Articular Index;* PASI*, Psoriasis Area and Severity Index;* VAS*, Visual Analogue Scale (0-100);* GH*, Global Health;* DAS*, Disease Activity Score;* HAQ*, Health Assessment Questionnaire;* TNF*, Tumour Necrosis Factor;* DMARDs*, Disease Modifying Anti-Rheumatic Drugs;* ns*, non-significant.*

Supplementary Table S4

		LYMPHO-MYELOID (3 PATIENTS)	DIFFUSE-MYELOID (11 PATIENTS)	PAUCI-IMMMUNE (10 PATIENTS)	p-values
BASELINE	Female % (n)	66.7% (2)	72.7% (8)	40.0% (4)	ns
	Age years (mean ± SD)	52.1 ± 22.5	47.8 ± 11.0	42.4 ± 12.2	ns
	Disease duration years (mean ± SD)	22.7 ± 23.0	4.5 ± 2.1	12.5 ± 12.5	ns
	ESR mm/hr (mean ± SD)	23.7 ± 2.1	25.9 ± 17.9	23.6 ± 20.1	ns
	CRP mg/l mean (mean ± SD)	2.0 ± 3.5	17.1 ± 27.9	10.9 ± 17.8	ns
	TJC/78 (mean ± SD)	36.0 ± 31.2	28.9 ± 20.9	38.1 ± 21.2	ns
	TJC/68 (mean ± SD)	31.3 ± 26.6	27.0 ± 18.3	33.9 ± 17.5	ns
	RAI (mean ± SD)	20.7 ± 12.3	16.9 ± 8.3	19.5 ± 10.1	ns
	SJC/76 (mean ± SD)	15.7 ± 6.7	11.6 ± 9.8	13.6 ± 11.3	ns
	SJC/66 (mean ± SD)	14.7 ± 8.0	11.1 ± 10.0	13.4 ± 11.4	ns
	SJC/44 (mean ± SD)	9.7 ± 6.8	9.7 ± 9.2	11.3 ± 9.2	ns
	Skin involvment, yes % (n)	66.7% (2)	72.7% (8)	80.0% (8)	ns
	PASI (mean ± SD)	3.2 ± 4.0	3.8 ± 4.0	5.9 ± 5.7	ns
	VAS tiredness (mean ± SD)	61.7 ± 2.3	60.4 ± 34.3	61.0 ± 28.8	ns
	VAS pain (mean ± SD)	76.0 ± 3.6	70.0 ± 26.4	66.7 ± 20.4	ns
	VAS GH Patient (mean ± SD)	83.0 ± 10.8	67.0 ± 30.2	68.6 ± 19.2	ns
	VAS GH Physician (mean ± SD)	68.3 ± 12.1	63.7 ± 12.6	60.0 ± 14.7	ns
	LIKERT Patient (mean ± SD)	4.0 ± 1.0	3.6 ± 0.7	3.9 ± 0.7	ns
	LIKERT Physician (mean ± SD)	4.0 ± 0	3.6 ± 0.7	3.4 ± 0.5	ns
	DAS (mean ± SD)	4.6 ± 1.3	4.2 ± 1.1	4.4 ± 1.3	ns
	HAQ (mean ± SD)	2.4 ± 0.6	1.9 ± 0.6	1.7 ± 0.6	ns
	Previous anti-TNF, yes % (n)	33.3% (1)	27.3% (3)	40.0% (4)	ns
	Current DMARDs, yes % (n)	66.7% (2)	81.8% (9)	50.0% (5)	ns
	Assigned to Anti-TNF, % (n)	66.7% (2)	72.7% (8)	60.0% (6)	ns
	Assigned to Ustekinumab,% (n)	33.3% (1)	27.3% (3)	40.0% (4)	ns

Table S4 Baseline clinical variables comparison between patients characterized by a lympho-myeloid (n=3), diffuse-myeloid (n=11) or pauci-immune (n=10) pathotype as previously defined in rheumatoid arthritis [14]. Briefly, immune-cells infiltrate was defined by immunohistochemistry (CD3 for T-cells, CD20 for B-cells, CD68 for macrophages and CD138 for plasma cells) and quantified using a semi-quantitative score (0-4) [14]. Accordingly, patients were categorised as lympho-myeloid if CD20 score ≥2 and/or CD138 score >2, diffuse-myeloid if CD68 sublining score ≥2, CD20 score ≤1 and CD138 score ≤2, and pauci-immune if CD68 sublining score <2 and CD3/CD20/CD138 score <1. 24/27 baseline synovial tissues were included in the analysis; 3 samples were classified as "ungraded" (absence of lining layer and/or necrotic tissue and/or absent of macrophages in the sublining). p-values were calculated using Kruskal Wallis or Fisher's exact test as appropriate.

SD, *Standard Deviation;* n, *number*; ESR, *erythrocyte sedimentation rate;* CRP, *C-Reactive Protein;* TJC, *Tender Joints Count;* SJC, *Swollen Joints Count;* RAI, *Ritchie Articular Index;* PASI, *Psoriasis Area and Severity Index;* VAS, *Visual Analogue Scale (0-100);* GH, *Global Health;* DAS, *Disease Activity Score;* HAQ, *Health Assessment Questionnaire;* TNF, *Tumour Necrosis Factor;* DMARDs, *Disease Modifying Anti-Rheumatic Drugs;* ns, *non-significant.*

Nerviani A, *et al. Ann Rheum Dis* 2021; 80:591–597. doi: 10.1136/annrheumdis-2020-218186

Supplementary Figure S2

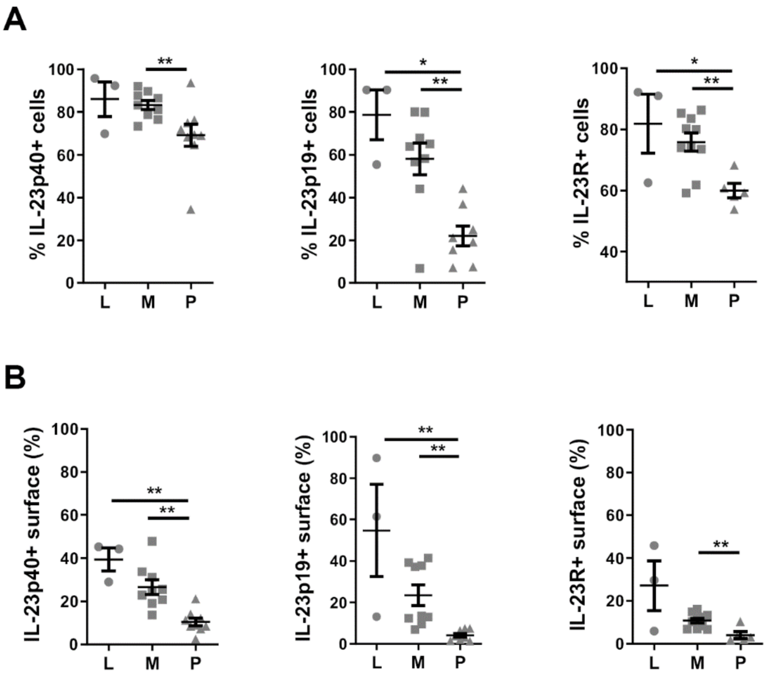

Figure S2 A, Distribution of the % of IL-23p40-, IL-23p19- and IL-23R-positive cells (of the total number of cells) according to the histological pathotypes at baseline. B, Distribution of the % of IL-23p40-, IL-23p19- and IL-23R-positive surface (of the total area) according to the histological pathotypes at baseline. L = **L**ympho-myeloid (n=3), M= diffuse-**M**yeloid (n=9-10), P= **P**auci-immune (n=5-9). Results are presented as mean ± standard deviation. *p < 0.05, **p < 0.01 as assessed by Kruskal–Wallis with Dunn's post-test.

Supplementary Table S5

	IL-23p40	IL-23p19	IL-23R
**Anti-TNF**	76.8 ± 14.0	43.2 ± 28.6	70.7 ± 14.2
**Ustekinumab**	78.0 ± 13.3	55.1 ± 27.8	72.1 ± 12.6

Table S5 IL-23p19, IL-23p40 and IL-23R baseline synovial protein expression levels stratified according to the biologic DMARD received afterwards (anti-TNF or ustekinumab). IL-23p19, IL-23p40 and IL-23R were detected by immunohistochemistry and quantified by Digital Image Analysis. Mean ± Standard Deviation of % of positive cells are represented. Anti-TNF: n=14-16; ustekinumab: n = 5-6.

Nerviani A, *et al. Ann Rheum Dis* 2021; 80:591–597. doi: 10.1136/annrheumdis-2020-218186

Supplementary Table S6

		IL-23p40		IL-23p19		IL-23R	
		r	p-values	r	p-values	r	p-values
BASELINE	ESR mm/hr	0.04	0.84, ns	0.02	0.93, ns	0.39	0.10, ns
	CRP mg/l	0.02	0.92, ns	0.15	0.53, ns	0.14	0.57, ns
	TJC/78	-0.14	0.54, ns	-0.09	0.70, ns	-0.26	0.28, ns
	TJC/68	-0.13	0.56, ns	-0.09	0.70, ns	-0.28	0.25, ns
	RAI	-0.02	0.91, ns	0.02	0.95, ns	-0.05	0.83, ns
	SJC/76	0.10	0.67, ns	0.21	0.37, ns	-0.31	0.19, ns
	SJC/66	0.08	0.73, ns	0.11	0.65, ns	-0.34	0.16, ns
	SJC/44	0.05	0.83, ns	-0.05	0.83, ns	-0.44	0.06, ns
	PASI	-0.13	0.61, ns	0.16	0.57, ns	0.13	0.64, ns
	VAS tiredness	0.13	0.56, ns	0.31	0.19, ns	-0.17	0.48, ns
	VAS pain	-0.21	0.34, ns	0.32	0.17, ns	-0.32	0.18, ns
	VAS GH Patient	-0.09	0.70, ns	0.31	0.19, ns	-0.33	0.17, ns
	VAS GH Physician	0.12	0.61, ns	0.14	0.54, ns	-0.18	0.45, ns
	LIKERT Patient	-0.52	0.01, *	0.05	0.84, ns	-0.63	0.004, **
	LIKERT Physician	0.08	0.73, ns	0.44	0.05, ns	-0.08	0.73, ns
	DAS	0.13	0.57, ns	0.09	0.70, ns	-0.16	0.51, ns
	HAQ	0.29	0.20, ns	0.38	0.10, ns	0.13	0.58, ns

Table S6 Correlations between IL-23p40/IL-23p19/IL-23R protein expression (as percentage of positive cells assessed by IHC) and baseline clinical variables. p-values were calculated using Spearman r test. *p < 0.05, **p < 0.01

ESR, *erythrocyte sedimentation rate;* CRP, *C-Reactive Protein;* TJC, *Tender Joints Count;* SJC, *Swollen Joints Count;* RAI, *Ritchie Articular Index;* PASI, *Psoriasis Area and Severity Index;* VAS, *Visual Analogue Scale (0-100);* GH, *Global Health;* DAS, *Disease Activity Score;* HAQ, *Health Assessment Questionnaire;* TNF, *Tumour Necrosis Factor;* DMARDs, *Disease Modifying Anti-Rheumatic Drugs;* ns, *non-significant.*

Nerviani A, *et al. Ann Rheum Dis* 2021; 80:591–597. doi: 10.1136/annrheumdis-2020-218186

Supplementary Figure S3

A

EARLY PsA		TOTAL POPULATION (21 PATIENTS)
	Female % (n)	57.1% (12)
	Age years (mean ± SD)	42.0 ± 14.4
	Disease duration months (mean ± SD)	4.9 ± 2.7
	ESR mm/hr (mean ± SD)	33.8 ± 21.8
	CRP mg/l mean (mean ± SD)	19.4 ± 19.3
	TJC/28 (mean ± SD)	7.8 ± 8.1
	SJC/28 (mean ± SD)	4.4 ± 3.4
	VAS tiredness (mean ± SD)	48.4 ± 28.4
	VAS pain (mean ± SD)	54.3 ± 24.2
	VAS GH Patient (mean ± SD)	63.0 ± 24.2
	VAS GH Physician (mean ± SD)	54.3 ± 25.7
	DAS/28 (mean ± SD)	4.7 ± 1.2
	HAQ (mean ± SD)	1.3 ± 0.9

B

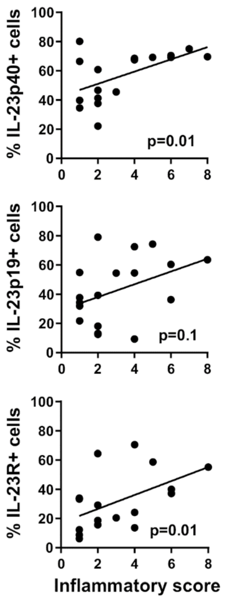

C

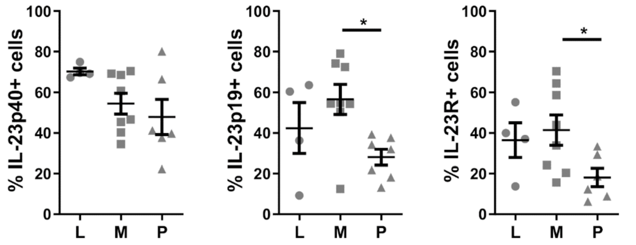

D

EARLY PsA		IL-23p40 r	IL-23p40 p-values	IL-23p19 r	IL-23p19 p-values	IL-23R r	IL-23R p-values
	ESR mm/hr	0.22	0.37, ns	0.20	0.42, ns	-0.02	0.93, ns
	CRP mg/l	0.23	0.37, ns	0.21	0.39, ns	-0.07	0.77, ns
	TJC/28	-0.32	0.19, ns	-0.36	0.13, ns	0.04	0.87, ns
	SJC/28	-0.04	0.88, ns	0.13	0.60, ns	0.32	0.2, ns
	VAS tiredness	-0.09	0.72, ns	-0.16	0.51, ns	0.30	0.23, ns
	VAS pain	0.10	0.69, ns	-0.24	0.31, ns	-0.10	0.69, ns
	VAS GH Patient	-0.35	0.16, ns	-0.22	0.37, ns	-0.01	0.98, ns
	VAS GH Physician	-0.08	0.76, ns	-0.24	0.33, ns	-0.11	0.65, ns
	DAS /28	-0.15	0.55, ns	-0.31	0.20, ns	-0.03	0.9, ns
	HAQ	-0.33	0.19, ns	-0.28	0.25, ns	-0.04	0.87, ns

Figure S3 IL-23 cytokines and receptor expression in early Psoriatic Arthritis. A, Demographic and clinical features of the early PsA cohort (n=21); B, Correlations between inflammatory scores and IL-23p40, IL-23p19 or IL-23R percentages of positive cells within the synovial tissue. p-values, calculated by Spearman's bivariate correlation analysis, are shown on each graph. C, Distribution of the % of IL-23p40-, IL-23p19- and IL-23R-positive cells (of the total number of cells) according to the histological pathotypes at baseline. L = **L**ympho-myeloid (n=4), M= diffuse-**M**yeloid (n=7-10), P= **P**auci-immune

(n=6-7). Results are presented as mean ± standard deviation. *$p < 0.05$ as assessed by Kruskal–Wallis with Dunn's post-test. D, Correlations between IL-23p40/IL-23p19/IL-23R protein expression (as percentage of positive cells assessed by IHC) and baseline clinical variables. p-values were calculated using Spearman r test. *SD, Standard Deviation; n, number; ESR, erythrocyte sedimentation rate; CRP, C-Reactive Protein; TJC, Tender Joints Count; SJC, Swollen Joints Count; VAS, Visual Analogue Scale (0-100); GH, Global Health; DAS, Disease Activity Score; HAQ, Health Assessment Questionnaire; ns, non-significant.*

Supplementary Figure S4

A

EARLY RA	TOTAL POPULATION (17 PATIENTS)
Female % (n)	70.6% (12)
Age years (mean ± SD)	43.4 ± 14.8
Disease duration months (mean ± SD)	4.4 ± 2.4
ESR mm/hr (mean ± SD)	27.9 ± 20.4
CRP mg/l mean (mean ± SD)	11.9 ± 12.5
TJC/28 (mean ± SD)	9.7 ± 5.5
SJC/28 (mean ± SD)	5.9 ± 2.4
VAS tiredness (mean ± SD)	29.1 ± 24.4
VAS pain (mean ± SD)	46.1 ± 29.1
VAS GH Patient (mean ± SD)	61.5 ± 24.4
VAS GH Physician (mean ± SD)	51.2 ± 23.9
RF+, yes % (n)	76.5% (13)
CCP+, yes % (n)	70.6% (12)
DAS/28 (mean ± SD)	5.0 ± 0.9
HAQ (mean ± SD)	1.1 ± 0.6

B

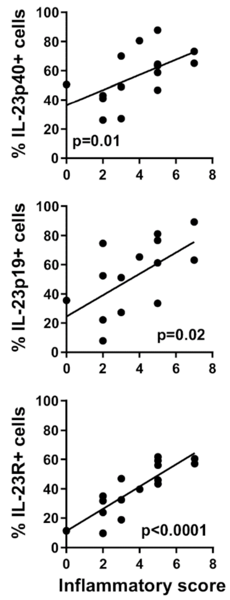

C

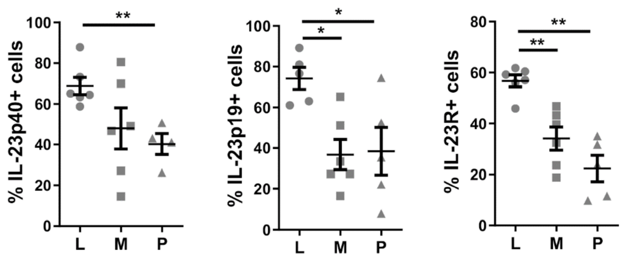

D

EARLY RA	IL-23p40 r	IL-23p40 p-values	IL-23p19 r	IL-23p19 p-values	IL-23R r	IL-23R p-values
ESR mm/hr	0.38	0.14, ns	0.34	0.19, ns	0.53	0.03, *
CRP mg/l	0.02	0.95, ns	0.17	0.52, ns	0.37	0.15, ns
TJC/28	0.40	0.13, ns	0.47	0.07, ns	0.28	0.27, ns
SJC/28	0.13	0.64, ns	0.32	0.23, ns	0.04	0.88, ns
VAS tiredness	0.39	0.13, ns	0.18	0.51, ns	0.29	0.25, ns
VAS pain	0.23	0.39, ns	0.63	0.01, *	0.46	0.07, ns
VAS GH Patient	0.06	0.83, ns	0.40	0.12, ns	0.30	0.24, ns
VAS GH Physician	0.24	0.36, ns	0.36	0.17, ns	0.27	0.29, ns
RF titer	0.06	0.83, ns	0.20	0.46, ns	0.01	0.97, ns
CCP titer	0.46	0.07, ns	0.34	0.19, ns	0.47	0.06, , ns
DAS /28	0.25	0.35, ns	0.51	0.05, *	0.33	0.19, , ns
HAQ	0.11	0.67, ns	0.22	0.41, ns	0.27	0.29, ns

Figure S4 IL-23 cytokines and receptor expression in early Rheumatoid Arthritis. A, Demographic and clinical features of the early arthritis RA cohort (n=17); B, Correlations between inflammatory scores and IL-23p40, IL-23p19 or IL-23R percentages of positive cells within the synovial tissue. p-values, calculated by Spearman's bivariate correlation analysis, are shown on each graph. C, Distribution of the % of IL-23p40-, IL-23p19- and IL-23R-positive cells (of the total number of cells) according to the histological pathotypes at baseline. L = **L**ympho-myeloid (n=6), M= diffuse-**M**yeloid (n=6), P= **P**auci-immune (n=5). Results are presented as mean ± standard deviation. *p < 0.05, **p < 0.01 as assessed

BMJ Publishing Group Limited (BMJ) disclaims all liability and responsibility arising from any reliance placed on this supplemental material which has been supplied by the author(s)

by Kruskal–Wallis with Dunn's post-test. D, Correlations between IL-23p40/IL-23p19/IL-23R protein expression (as percentage of positive cells assessed by IHC) and baseline clinical variables. $*p < 0.05$, p-values were calculated using Spearman r test. *SD, Standard Deviation; n, number; ESR, erythrocyte sedimentation rate; CRP, C-Reactive Protein; TJC, Tender Joints Count; SJC, Swollen Joints Count; VAS, Visual Analogue Scale (0-100); GH, Global Health; DAS, Disease Activity Score; HAQ, Health Assessment Questionnaire; ns, non-significant.*
